# Multimodal Deep Learning for Prognosis Prediction in Renal Cancer

**DOI:** 10.3389/fonc.2021.788740

**Published:** 2021-11-24

**Authors:** Stefan Schulz, Ann-Christin Woerl, Florian Jungmann, Christina Glasner, Philipp Stenzel, Stephanie Strobl, Aurélie Fernandez, Daniel-Christoph Wagner, Axel Haferkamp, Peter Mildenberger, Wilfried Roth, Sebastian Foersch

**Affiliations:** ^1^ Institute of Pathology, University Medical Center Mainz, Mainz, Germany; ^2^ Institute of Computer Science, Johannes Gutenberg University Mainz, Mainz, Germany; ^3^ Department of Diagnostic and Interventional Radiology, University Medical Center Mainz, Mainz, Germany; ^4^ Department of Urology and Pediatric Urology, University Medical Center Mainz, Mainz, Germany

**Keywords:** artificial intelligence, deep learning, pathology, prognosis prediction, radiology, renal cancer

## Abstract

**Background:**

Clear-cell renal cell carcinoma (ccRCC) is common and associated with substantial mortality. TNM stage and histopathological grading have been the sole determinants of a patient’s prognosis for decades and there are few prognostic biomarkers used in clinical routine. Management of ccRCC involves multiple disciplines such as urology, radiology, oncology, and pathology and each of these specialties generates highly complex medical data. Here, artificial intelligence (AI) could prove extremely powerful to extract meaningful information to benefit patients.

**Objective:**

In the study, we developed and evaluated a multimodal deep learning model (MMDLM) for prognosis prediction in ccRCC.

**Design, Setting, and Participants:**

Two mixed cohorts of non-metastatic and metastatic ccRCC patients were used: (1) The Cancer Genome Atlas cohort including 230 patients and (2) the Mainz cohort including 18 patients with ccRCC. For each of these patients, we trained the MMDLM on multiscale histopathological images, CT/MRI scans, and genomic data from whole exome sequencing.

**Outcome Measurements and Statistical Analysis:**

Outcome measurements included Harrell’s concordance index (C-index) and also various performance parameters for predicting the 5-year survival status (5YSS). Different visualization techniques were used to make our model more transparent.

**Results:**

The MMDLM showed great performance in predicting the prognosis of ccRCC patients with a mean C-index of 0.7791 and a mean accuracy of 83.43%. Training on a combination of data from different sources yielded significantly better results compared to when only one source was used. Furthermore, the MMDLM’s prediction was an independent prognostic factor outperforming other clinical parameters.

**Interpretation:**

Multimodal deep learning can contribute to prognosis prediction in ccRCC and potentially help to improve the clinical management of this disease.

**Patient Summary:**

An AI-based computer program can analyze various medical data (microscopic images, CT/MRI scans, and genomic data) simultaneously and thereby predict the survival time of patients with renal cancer.

## Introduction

Clear-cell renal cell carcinoma (ccRCC) is the most common type of kidney cancer and more than 175,000 patients die from this entity each year (1). In contrast to other tumor types, there is no clearly defined set of biomarkers used in clinical routine. This might be partly because ccRCC development seems to be driven by a multitude of interacting metabolic pathways and regulated by complex epigenetic programs (2). Clinical management of ccRCC usually involves various specialties including urology, radiology, oncology, pathology, and others. This results in a vast amount of medical data on each patient, such as CT/MRI scans, histopathological images, and other clinical information. There are several clinical tools for prognosis prediction in ccRCC, such as the UCLA integrated Staging System (UISS) (3) or the risk model of the International Metastatic RCC Database Consortium (IMDC) (4). Heng et al. for example developed a score, which consists of various clinical parameters such as Karnofsky performance status, hemoglobin, corrected calcium, and others. With this strategy, they were able to achieve an overall C-index of 0.73 in the prognosis prediction of 645 metastatic RCC (4). But while prognostic clinical nomograms might be helpful, they can be cumbersome to use and often only incorporate a selection of the available information—both of which potentially limit their performance. Here, artificial intelligence (AI) and machine learning (ML) could prove extremely helpful to utilize these highly complex data to predict clinically relevant outcomes such as survival or therapy response.

AI and ML are increasingly being applied to various medical problems achieving highly promising results in ophthalmology (5), radiology (6), cardiology (7), and others—even surpassing human level performance in some cases (8). For pathological tasks for example, we were able to predict the molecular subtype of muscle-invasive bladder cancer from conventional histopathological slides alone using deep learning (DL) (9). We also used a similar approach for prognosis prediction in soft tissue sarcoma (STS) (10). But while it is technically feasible, there are very few studies so far evaluating the use of multimodal input for training of AI and DL models (11). Thus, we developed a comprehensive DL pipeline and utilized multiscale conventional histopathological images together with CT/MRI images and genomic data to predict survival in patients with ccRCC.

## Materials and Methods

### Patient Cohorts

Two cohorts were utilized in which patients with ccRCC were included. The first cohort served as the basis for training of the neural network and validation to determine performance metrics. It consisted of all patients of the KIRC TCGA (Kidney renal clear cell carcinoma of the Cancer Genome Atlas) cohort for which the diagnostic H&E (hematoxylin & eosin) stained whole slide as well as radiological images were available. These were downloaded for 230 patients through the GDC portal (https://portal.gdc.cancer.gov/) as well as from the cancer imaging archive (https://www.cancerimagingarchive.net/). All initial pathology reports, clinicopathological and survival data (disease-specific survival, DSS) as well as the ten most frequent mutations/copy number alterations in our cohort were gathered from www.cbioportal.org. A comprehensive quality assessment excluded histopathological slides with large folds, no tumor tissue, and/or where the image was out of focus. Whenever possible computer tomography (CT) scans with nephrogenic or late systemic arterial phase were used. In a subset of patients, only magnet resonance imaging (MRI) scans were available. In this case T1-weighted sequences were used when possible. A second, mono-center cohort of 18 patients was generated as an additional external test set (the Mainz cohort). These patients were diagnosed between 2011 and 2015 at the University Medical Center Mainz. Retrospective use of these and other patients’ data and material for research purposes was approved by the ethical committee of the medical association of the State of Rhineland-Palatinate [Ref. Nos. 837.360.16 (10679) and 837.031.15(9799)] and results were generated after 2-fold pseudonymization of the cohort. We settled on this relatively low number of patients to ensure high quality of the radiologic, pathologic, and clinical follow up data. Tumor staging, grading, and treatment for these patients was carried out according to the appropriate guidelines in place at that time (i.e., ISUP). All experiments were in accordance with the Declaration of Helsinki (**Supplementary Tables 1**, **2**).

### Scanning and Preprocessing

TCGA whole slide images (WSIs) were digitalized at various institutions participating in the TCGA consortium. Slides from the second cohort were scanned using a Hamamatsu Nanozoomer Series scanner (Hamamatsu Photonics, Hamamatsu, Japan) at 40-fold magnification. This translated to a resolution of 0.23 µm/pixel. Slides were thoroughly evaluated for routine diagnostics by a board-certified pathologist and annotated by the project team blinded to any of the target variables. Annotation describes the process in which a polygonal region of interest was drawn around the tumor area. Various tissue aspects were considered (i.e., necrosis, etc.). Image tiles (520 px^2^) from two magnification levels (level 5 ≈ 10× magnification and level 10 ≈ 5× magnification) were then generated from these annotations. All tiles were normalized to an external reference image of a different dataset using structure preserving color normalization (SPCN) as proposed by Vahadane et al. (12) and Anand et al. (13). CT/MRI scans were also gathered at the respective institutions participating in the TCGA consortium. CT scans from the second cohort were generated using 64- or 128-section CT systems (Philips, Eindhoven, Netherlands) and 1.5T MRI scanner (Siemens, Forchheim, Germany). Scans were thoroughly evaluated for routine diagnostics by a board-certified radiologist and annotated by the project team blinded to any of the target variables. For each 3D volume, three images were extracted showing the maximum tumor diameter (one transversal plane, one sagittal plane and one coronal plane). Examples for annotation, tiling, normalization, and augmentation can be found in **Supplementary Figures 1A–C**).

### Novel Deep Learning Pipeline

A new, comprehensive, multimodal deep learning model (MMDLM) was developed consisting of one individual 18-layer residual neural network (ResNet) per image modality (resulting in up to three ResNets) and a dense layer for genomic data. This particular architecture was chosen to compromise between model depth and computational time. After this, the network outputs were concatenated by an attention layer, which weights every output due to its importance for the given task and passed through a final fully connected network, followed by either C-index calculation (see below) or binary classification (5-year disease-specific survival status (5YSS)). The 5YSS includes all patients who either lived longer than 60 months or who passed away within five years after diagnosis. All patients for who the follow-up time was shorter than five years were not included in this analysis, as one cannot be certain, whether these patients would have survived five years or longer. Unimodal training was performed by muting all other inputs and initializing ResNet weights on pretrained image net weights. Multimodal training was then carried out using the pretrained weights of the unimodal training. A total of 200–400 epochs were trained, and the best model was chosen when training and validation curves stopped converging. Standard Cox loss was used as loss function and cross-entropy loss was used as loss function for binary classification. Cox loss is defined as


$$l_{cox}(\theta ):=-\sum_{i:E_{i}=1}\left({\hat{h}}_{\theta }(x_{i})-\log\sum_{j:T_{j}>T_{i}}e^{{\hat{h}}_{\theta }(x_{j})}\right)$$

Where T_i_ represents the survival time, E_i_ the censored event status, x_i_ the data for each patient, and ${\hat{h}}_{\theta }$ the neural network model trained (14). Stochastic gradient descent was used as optimizer, learning rate was set at 0.004, momentum was set at 0.9, batch size was 32. Training was performed with a customized data loader which generated random combinations of one histopathologic image at level 5, one at level 10 and one radiologic image for each patient. Genomic data could optionally be included, but we limited the number to the 10 most frequent mutations, not to make the model overly complex. Validation was performed on a patient level using the Cartesian product of primary fixed image combinations to make results more comparable. Classification markup was performed using our previously reported sliding-window approach after training of an unmodified 18-layer ResNet (9). Class activation maps (CAMs) were established as recently described (15).

### Statistical Analysis

Training and validation on the TCGA cohort were performed using full k-fold cross validation (CV) on a patient level (6-fold CV for C-index prediction and 12-fold CV for binary classification). Metrics included C-index, recall (sensitivity), true negative rate (specificity), precision, area under the curve (AUC) of the receiver operating characteristic (ROC) and also the precision recall curve (PRC). Concordance index (C-index) was calculated as implemented by the lifelines package (16). In short, it is a measurement of the ability of a model to rank each patient according to their actual survival times based on individual risk scores. We used the C-index implementation by Davidson-Pilon et al. (16) as the number of censored events was not unreasonably high and as it was the easiest to integrate into our setup. For the 5YSS all patients surviving longer than 5 years were compared to all patients who died within the first 5 years. Patients lost to follow up within the first five years were not included in these analyses. The mean AUC of ROC either of multiple classes or as a summary of cross validation for each individual class was calculated using micro- and macro-averaging (17). For each analysis, the values’ distribution was tested. Paired t-test was used when two individual groups with normal distribution of paired experiments were analyzed. One-sample t-test was used to compare column means to a single value. Repeated measures (RM) one-way ANOVA with *post-hoc* Tukey HSD to correct for multiple comparisons was used when more than two groups with normal distribution were compared. Log-rank test was used for comparison of two or more survival curves. Univariable and multivariable Cox regression was used for prognosis analyses after checking proportionality using scaled Schoenfeld residuals. If not indicated otherwise, ± standard deviation (SD) is given. Differences in the compared groups were considered statistically significant when P values were smaller than 0.05 (p ≥0.05: ns, p = 0.01–0.05:*, p = 0.001–0.01:**, p = 0.0001–0.001:***, p <0.0001:****). For annotations and image preprocessing of the histopathologic slides, QuPath open source software (18) was used. For annotation and image processing of the radiologic volumes, Mango (19) and 3D Slicer (20) were used. All deep learning experiments were done in Python using PyTorch/fast.ai or TensorFlow/Keras. Statistical analysis was done using Graph Pad Prism or R. Some images were created with BioRender.com. Our algorithms were developed utilizing open access material and tutorials, such as PyImageSearch by Adrian Rosebrock, “Practical Deep Learning for Coders” by Jeremy Howard, and others. Code samples, etc. might be provided within collaboration with the project team. Please contact the corresponding author.

## Results

**Figure 1:**
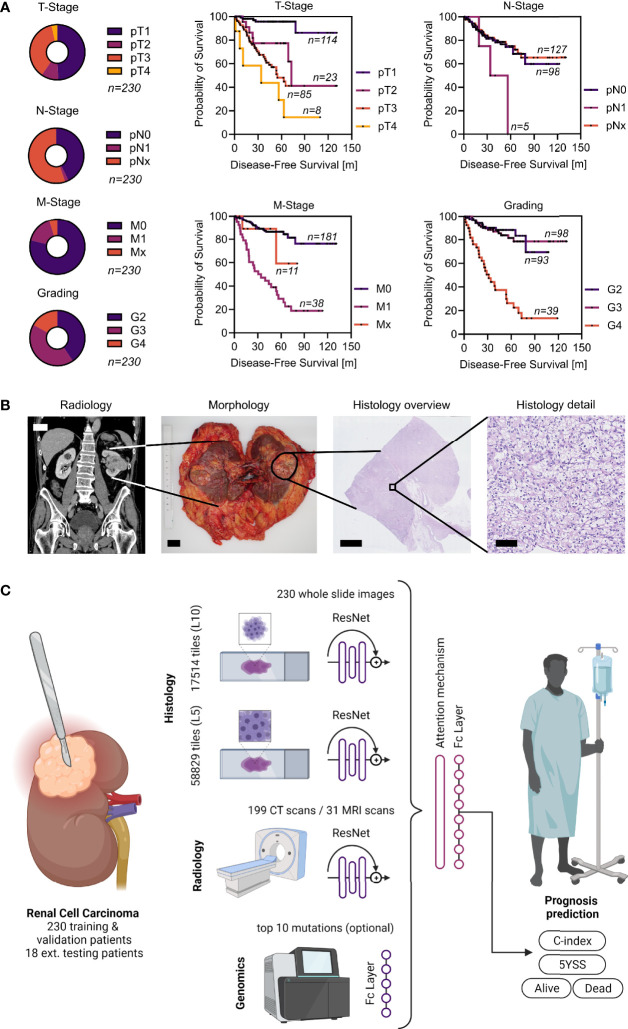
Patient cohort, clinical example, and overview of the MMDLM. **(A)** Characteristics of the TCGA cohort. **(B)** Clinical example of a typical ccRCC case. CT (scalebar 5 cm), macroscopic (scalebar 2 cm), as well as histologic tumor appearance (scalebars 5 mm and 100 µm) are displayed. **(C)** Schematic overview of the model. Created with BioRender.com.

Clinicopathological features of the TCGA cohort can be found in **Figure 1A**. A total of 58,829 tiles at level 5 and 17,514 tiles at level 10 were generated from 230 whole slide images for the training and validation experiments. Approximately 199 CT scans as well as 31 MRI scans from the same cohort were used to generate a total of 690 coronal, sagittal and transversal images. A typical example of a ccRCC case and a scheme of the MMDLM is displayed in **Figures 1B, C**.

**Figure 2:**
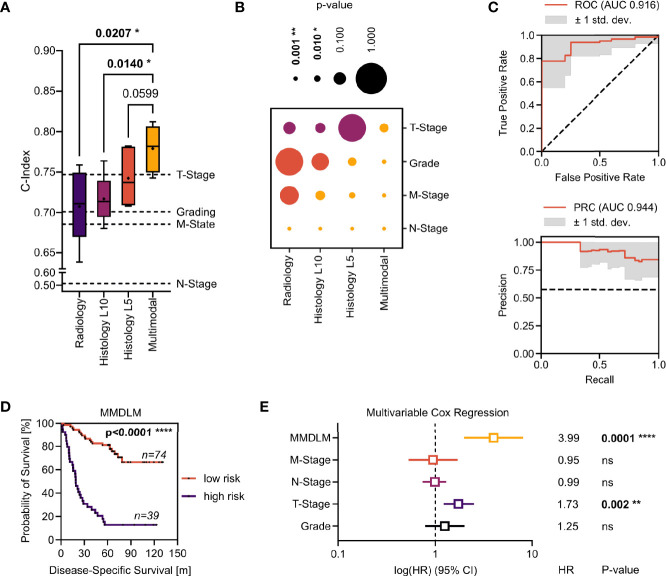
Evaluation of the MMDLM for prognosis prediction in ccRCC. **(A)** C-index distribution of 6-fold cross validation. Dotted lines represent the C-index of the respective clinical attribute (Grading, T-Stage, N-Stage, M-Stage) of the whole cohort. RM one-way ANOVA with *post-hoc* Tukey HSD to correct for multiple comparisons was used to compare the groups. **(B)** P-value matrix of one-sample t test of each modality vs. each risk factor (yellow: significantly. higher, orange: higher, purple: lower). **(C)** Mean ROC (top) and PR curve (bottom) of 12-fold cross validation. **(D)** Kaplan–Meier-Curve after stratification according to 5YSS by the MMDLM. **(E)** Forrest plot of multivariable Cox regression. HR, hazard ratio; CI, Confidence interval; Ns, not significant. *p = 0.01–0.05, **p = 0.001–0.01, ****p < 0.0001

First, we wanted to establish a baseline of the prognosis prediction capabilities of each imaging modality alone. To this end we calculated the C-index, which is a measurement of the ability of a model to rank each patient according to their actual survival times based on individual risk scores. Using unimodal training on radiological data yielded a mean C-index of 0.7074 ± 0.0474 with a maximum of 0.7590. Training only on histopathological image tiles our model achieved a mean C-index of 0.7169 ± 0.0296 with a maximum of 0.7638 (level 10) and a mean C-index of 0.7424 ± 0.0339 with a maximum of 0.7821 (level 5), respectively. Next, we wanted to investigate, whether the combination of different imaging modalities would improve prognostication in ccRCC. When combining conventional histopathological input with CT and MRI images, the mean C-index increased to 0.7791 ± 0.0278 with a maximum of 0.8123. There was a significant difference when compared to C-index of training only on radiologic images (p-value = 0.0207) and histopathologic tiles (p-value = 0.0140) (**Figure 2A**). Next, we wanted to investigate how the uni- and multimodal deep learning models performed, when compared to known prognostic factors in renal cell carcinoma. Cox proportional hazard (CPH) models were used to calculate the C-indices for histopathological grading (0.7010), T-Stage (0.7470), N-Stage (0.5140), and M-Stage (0.6850). Strikingly, only the MMDLM was significantly better than all independent prognostic factors (**Figure 2B**).

Since the C-index cannot be applied to an individual patient and thus might prove difficult to be translated into clinical decision making, we investigated the possibility to predict the 5-year survival status (5YSS) using a MMDLM and binary classification. A total of 113 patients could be included in these analyses. Here accuracy reached 83.43% ± 11.62% with a maximum of 100% upon 12-fold cross validation. This was higher, when compared to unimodal approaches, however this did not reach statistical significance. AUC of the ROC was 0.916 ± 0.105 with a maximum of 1.0. AUC of the PR curve was 0.944 ± 0.075 with a maximum of 1.0 (**Figure 2C**). Dividing the cohort according to the MMDLM’s prediction (“Alive” vs. “Dead”) into low- and high-risk patients showed a highly significant difference in the survival curves (**Figure 2D**). This was also true when only non-metastasized (M0) or metastasized (M+) patients were evaluated (**Supplementary Figure 2**). To compare the MMDLM’s prediction with the known risk factors described above, we performed multivariable regression analyses. Here only T-Stage and MMDLM’s prediction showed to be independent, significant prognostic factors with the MMDLM displaying the highest hazards ratio of almost 4 (**Figure 2E**).

**Figure 3:**
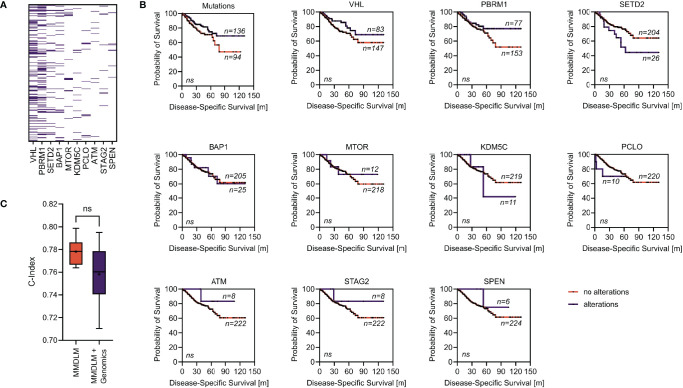
Addition of genomic information does not improve the MMDLM. **(A)** Distribution of the ten most frequent mutations/CNA in our cohort. **(B)** Survival stratified according to mutational status (alterations/no alterations) of the genes selected in panel **(A)**. **(C)** C-Index distribution using a MMDLM without and with the mutational status included. Ns, not significant.

To investigate whether the addition of genomic data could further improve our image-based prognosis prediction, we compared the performance of the MMDLM with and without training on the top ten mutations/copy number alterations (CNA) found in our cohort (**Figure 3A**). Interestingly, there was no improvement by adding this type of information to the training process. Looking at all alterations together or each alteration separately, none was able to show a statistically significant difference in survival of patients with ccRCC (**Figures 3B, C**).

Since we only trained and evaluated on the TCGA dataset thus far, we wanted to investigate how the MMDLM would perform on an additional external test set. This consisted of 18 patients representing 9.3% of the training set for C-index calculation and 17.6% for binary classification. Mean C-index reached 0.799 ± 0.060 with a maximum of 0.8662. Accuracy averaged at 79.17% ± 9.8% with a maximum of 94.44%. AUC of the ROC was 0.905 ± 0.073 with a maximum of 1.0. AUC of the PR curve was 0.956 ± 0.036 with a maximum of 1.0. All performance measures were not significantly different from those achieved during cross validation (CV) (**Supplementary Figure 3**).

**Figure 4:**
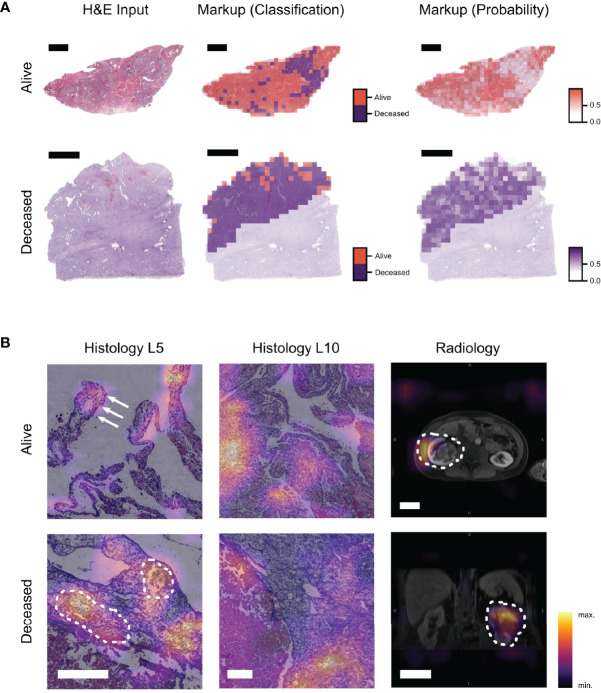
Visualization techniques show image regions important for the prediction and their contribution to the MMDLM. **(A)** Example of a visualization approach to display the classification result of a unimodal histopathology model (ResNet18—Level 5). The input WSI as well as two different markup images are displayed. Markup all denotes the distinction between tiles classified as alive or deceased. Markup class denotes the prediction certainty within the majority class (scalebar top row: 4 mm, scalebar bottom row: 5 mm). **(B)** CAMs of the MMDLM are shown. Different features associated with low-risk (alive) and high-risk (deceased) are highlighted. In the low-risk example, clear cell morphology as well as papillary tumor appearance (arrows) can be observed. In the high-risk example, tumor vasculature and bleeding can be observed (dotted line) (scalebar histology: 250 µm, scalebar radiology: 5 cm).

Lastly, we aimed at increasing the transparency of our model by visualizing the image features for each modality that were most relevant to the model’s prediction. We used a sliding window approach to visualize unimodal classification WSIs (**Figure 4A**). We established class activation maps (CAMs) using the CV fold with the highest C-index prediction, consisting of 17,550 image combinations. Investigating these image combinations of all patients of this fold, a first descriptive screening analysis of representative CAMs revealed histopathologic (such as tumor vasculature, hemorrhage, and necrosis) and radiologic (such as tumor volume) features which were most important to the model to make its prediction (**Figure 4B**).

## Discussion

Diagnosis and treatment of ccRCC remains a clinical challenge—especially in metastasized cases. For both non-metastasized and metastasized patients, prognostic tools exist such as UISS and IMDC, but there is still room for improvement. In our study, we propose a MMLDM, which could be a valuable alternative and/or addition to existing tools in both M0/M+ patients. While methods of AI are increasingly being used in various medical domains, their combination across different modalities has only rarely been explored (21). This is particularly surprising as such combination efforts are already being developed and deployed in non-medical fields such as autonomous driving and others. Furthermore, the few multimodal or “fusion” approaches applied to medical problems consistently showed a boost in accuracy of up to 27.7% when compared over single modality models for the same task (22). However, most of this work is limited to the integration of low-level clinical features with one type of imaging data to make a certain diagnosis. Here we describe three major improvements over most previous studies. (I) A variety of comprehensive histopathologic and radiologic imaging techniques together with genetic information derived from whole exome sequencing were integrated in our model. This mirrors the clinical decision-making process (i.e., during interdisciplinary tumor boards) and was done to ensure, that as much of the relevant information was utilized as possible. (II) Target variable was not the diagnosis of a certain tumor entity but rather the prognosis of the patients. This is particularly relevant in renal cancer as there is an urgent need for reliable prognostic biomarkers in this entity. Our integrative approach could be used to distinguish between low- and high-risk patients, who would be more suitable for intensified treatment and/or surveillance. Interestingly, the addition of genomic data did not improve the image-based multimodal approach—highlighting the fact that mutations/CNA are of less prognostic value in our cohort. This might be because ccRCC is highly dependent on mutations that are very common in this tumor type. (III) We also used additional visualization techniques to highlight image features which were most relevant to our model.

Liu et al. used photographs together with clinical data to classify skin lesions and showed that the top-1-accuracy of their deep learning system was even slightly better than the one of trained dermatologists (23). However, to achieve this accuracy the group had to use data from over 15,000 patients, which might not be easily accessible for every clinical question. Furthermore, while the authors show how training only on images decreases the model’s performance it is unclear how the model would have performed on clinical data alone. By using CAMs, we were able to investigate image features associated with prognosis, although only in a descriptive fashion thus far. A recent publication by Ning et al. uses convolutional neural networks (CNNs) for feature extraction on radiologic and pathologic data, and combines these features with genomic data for prognosis prediction in ccRCC (24) with similar results. However, in the study by Ning et al. it remains unclear how and which image features were selected and how the model would perform on a true external test set. Of course, there are limitations to our approach as well. For example the comparison between other clinical tools, which include clinical data such as performance status, calcium levels, etc. are missing. So a head to head comparison with IMDC or UISS scores is necessary to determine superiority of our MMDLM. Furthermore, the size of the external validation is rather small, and additional studies are needed to ensure generalizability of our approach.

## Data Availability Statement

The datasets presented in this article are not readily available because of institutional requirements and general data protection regulation. The WSI for the TCGA Dataset are publicly available from the Genomic Data Commons Data Portal. Requests to access the datasets should be directed to sebastian.foersch@unimedizin-mainz.de.

## Ethics Statement

The studies involving human participants were reviewed and approved by the ethical committee of the medical association of the State of Rhineland-Palatinate [Ref. No. 837.360.16(10679) and 837.031.15(9799)]. Written informed consent for participation was not required for this study in accordance with the national legislation and the institutional requirements.

## Author Contributions

Conception and design: SF, SSc, A-CW. Acquisition of data: SF, SSc, A-CW, FJ, CG, PS, SSt, AH, and PM. Analysis and interpretation of data: SF, SSc, A-CW, FJ, CG, PS, SSt, D-CW, PM, and WR. Drafting of the manuscript: SF and SSc. Critical revision of the manuscript for important intellectual content: A-CW, FJ, PS, AH, PM, and WR. Statistical analysis: SF, SSc, A-CW, and CG. Obtaining funding: SF. Administrative, technical, or material support: AF. Supervision: SF and WR. All authors contributed to the article and approved the submitted version.

## Funding

This work was supported by the Federal Ministry of Education and Research (16SV8167), the Stage-I-Program of the University Medical Center Mainz, the Mainz Research School of Translational Biomedicine (TransMed), and the Manfred-Stolte-Foundation.

## Conflict of Interest

The authors declare that the research was conducted in the absence of any commercial or financial relationships that could be construed as a potential conflict of interest.

## Publisher’s Note

All claims expressed in this article are solely those of the authors and do not necessarily represent those of their affiliated organizations, or those of the publisher, the editors and the reviewers. Any product that may be evaluated in this article, or claim that may be made by its manufacturer, is not guaranteed or endorsed by the publisher.

## Supplementary Material

Supplementary Figure 1 Data preprocessing. **(A)** Preprocessing of the input WSI (top) included annotation (middle) and tiling into level 5 tiles (bottom)(scalebar 2 mm). **(B)** Input tiles (top) were normalized (middle) to a reference image not from the cohorts investigated (scalebar 250 µm) and augmented (bottom) during training. **(C)** Preprocessing of the radiologic data included export of a coronal, transversal, and sagittal image with the largest tumor diameter (“pseudovolume”). Icon modified from CFCF, CC BY-SA 3.0, https://commons.wikimedia.org/w/index.php?curid=33285529.

Supplementary Figure 2 Survival of MMDLM low- and high-risk cases according to M0/M+. **(A)** Kaplan-Meier-Curve after stratification according to 5YSS by the MMDLM for M0 patients. **(B)** Kaplan-Meier-Curve after stratification according to 5YSS by the MMDLM for M+ patients.

Supplementary Figure 3 Evaluation of the MMDLM on an additional external test cohort. **(A)** Frequency distribution of the additional test cohort (the Mainz cohort) is similar to the TCGA cohort. **(B)** Mean C-index is not significantly different between the validation and the additional external test set. **(C)** Accuracy is not significantly different between the validation and the external test set. **(D)** ROC and PR curves of the external test set. Ns, not significant.

Supplementary Table 1 TCGA cohort.

Supplementary Table 2 Additional external test cohort (the Mainz cohort).
