# Supplementary figures and images for: Multimodal Deep Learning for Prognosis Prediction in Renal Cancer
Source: Front Oncol. 2021 Nov 24;11:788740. doi: 10.3389/fonc.2021.788740 (PMC8651560; doi:10.3389/fonc.2021.788740)

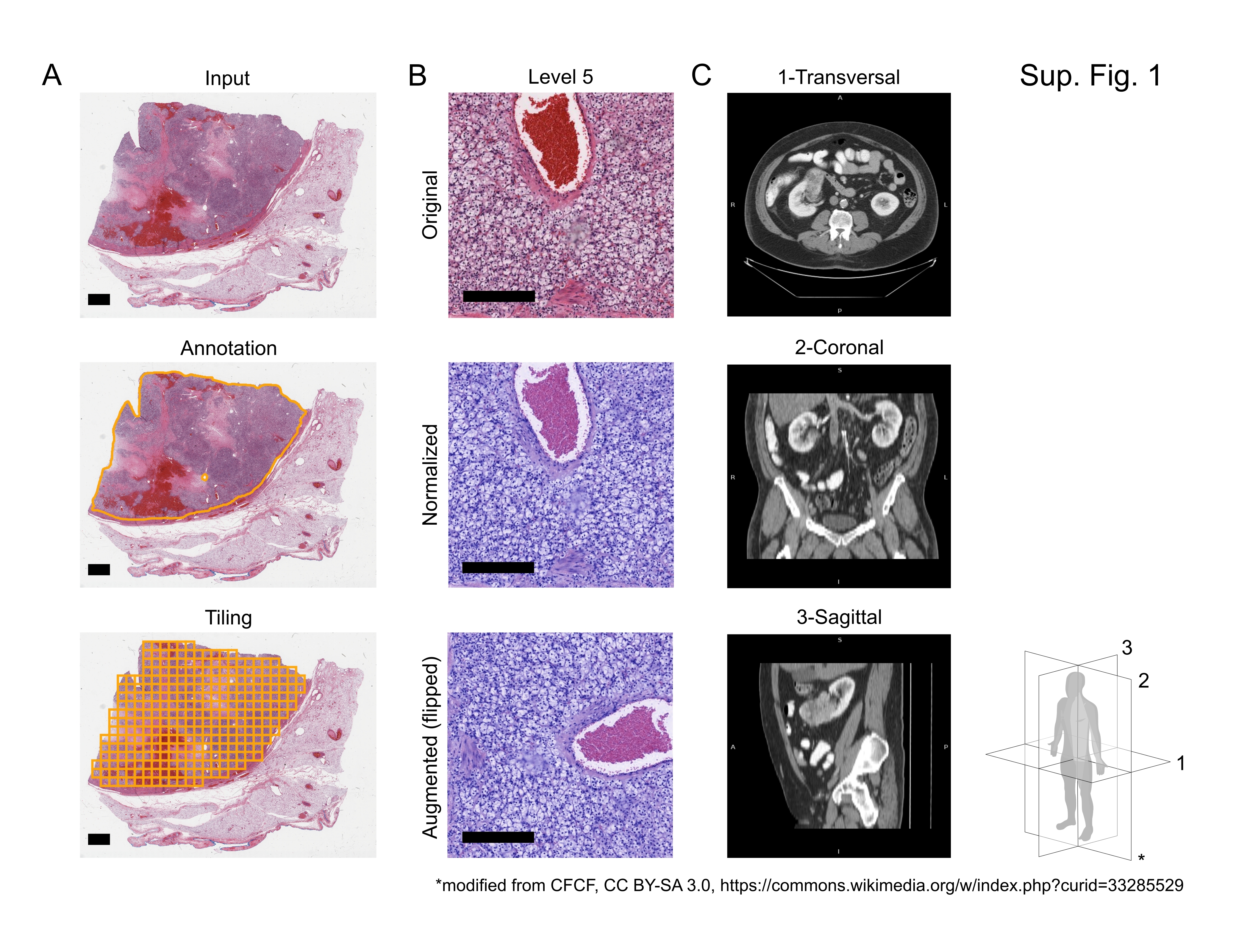

Supplement: Supplementary Figure 1 — Data preprocessing. (A) Preprocessing of the input WSI (top) included annotation (middle) and tiling into level 5 tiles (bottom)(scalebar 2 mm). (B) Input tiles (top) were normalized (middle) to a reference image not from the cohorts investigated (scalebar 250 µm) and augmented (bottom) during training. (C) Preprocessing of the radiologic data included export of a coronal, transversal, and sagittal image with the largest tumor diameter (“pseudovolume”). Icon modified from CFCF, CC BY-SA 3.0, https://commons.wikimedia.org/w/index.php?curid=33285529. [file Image_1.jpeg]

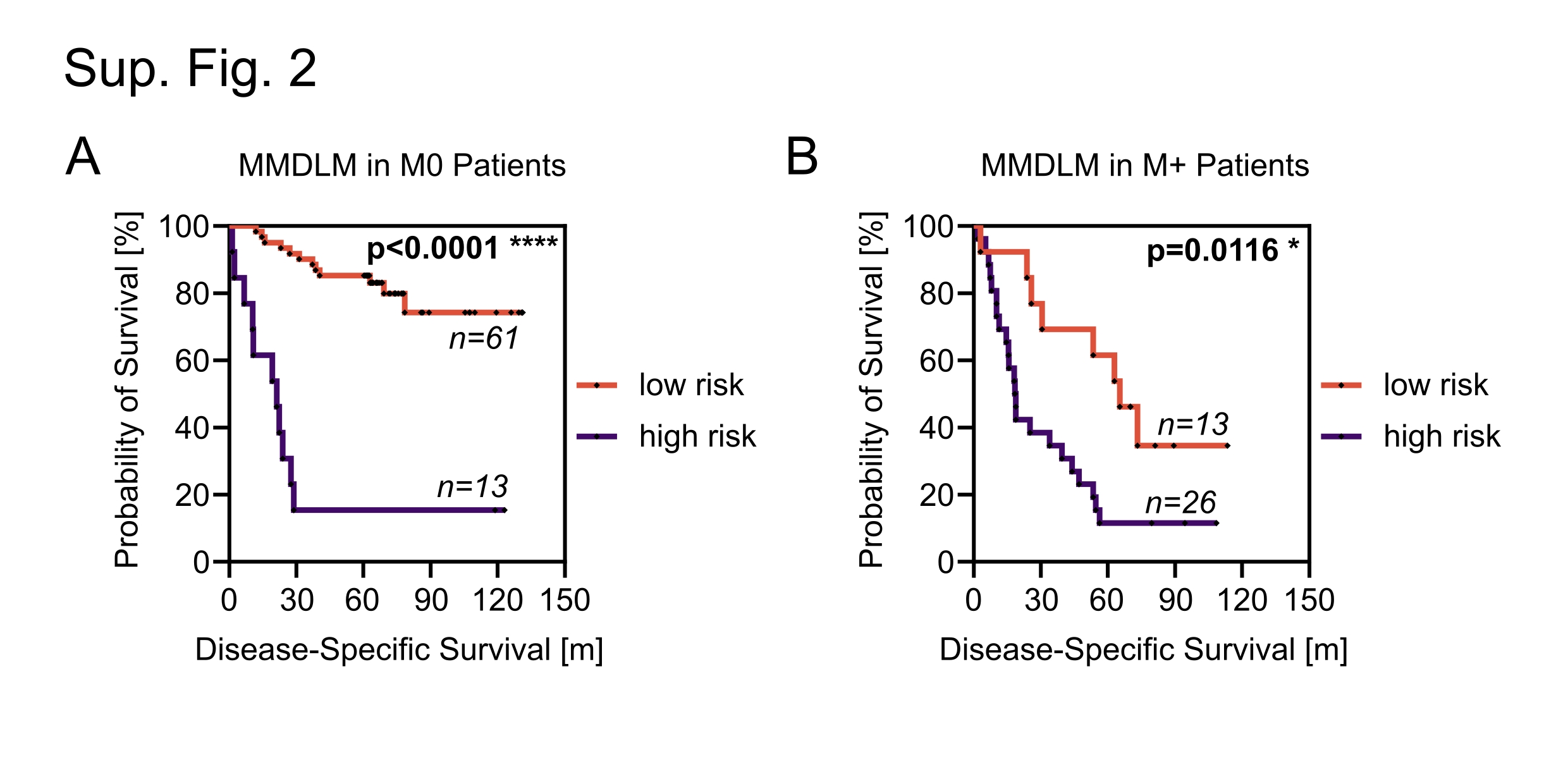

Supplement: Supplementary Figure 2 — Survival of MMDLM low- and high-risk cases according to M0/M+. (A) Kaplan-Meier-Curve after stratification according to 5YSS by the MMDLM for M0 patients. (B) Kaplan-Meier-Curve after stratification according to 5YSS by the MMDLM for M+ patients. [file Image_2.jpeg]

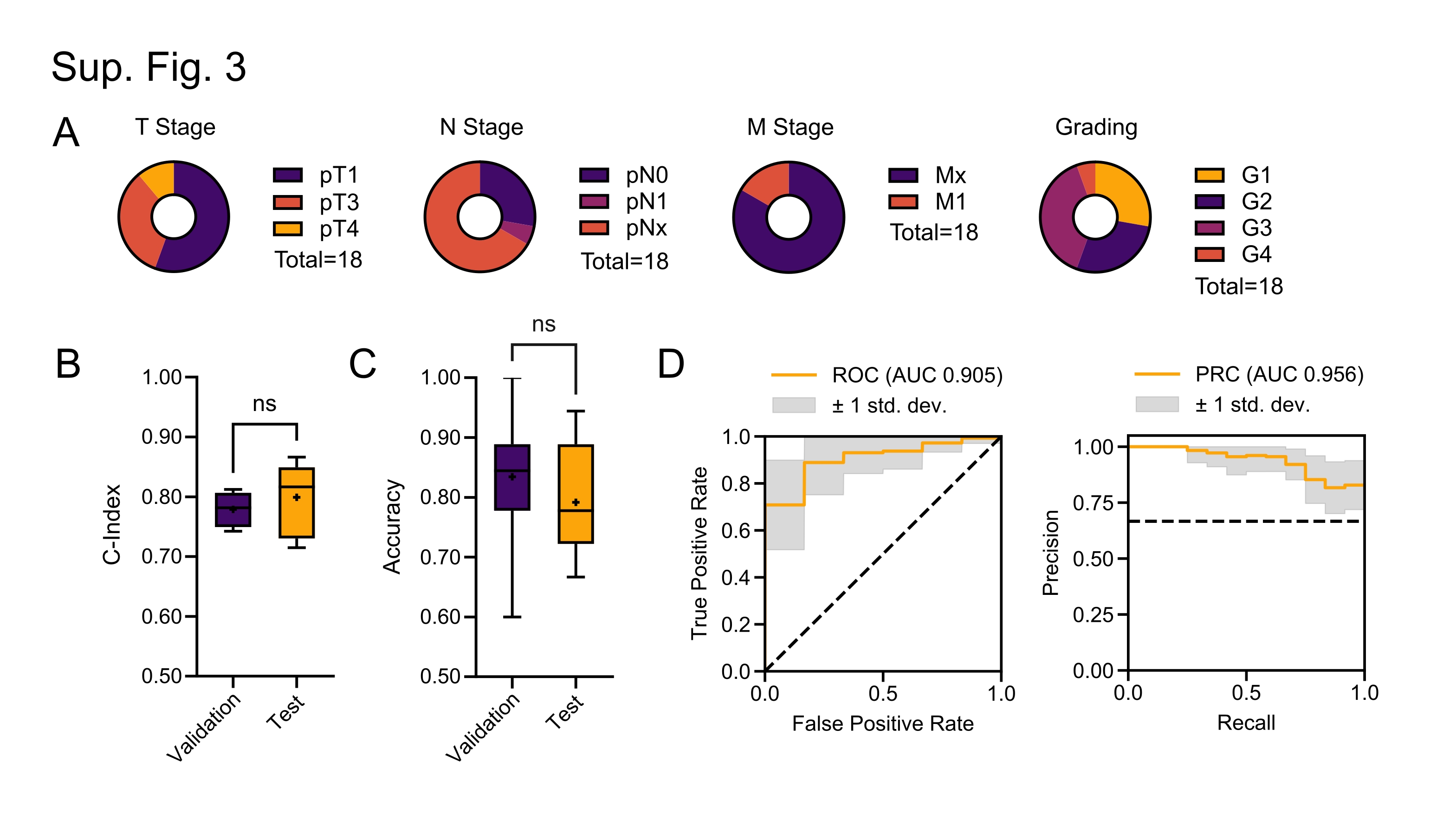

Supplement: Supplementary Figure 3 — Evaluation of the MMDLM on an additional external test cohort. (A) Frequency distribution of the additional test cohort (the Mainz cohort) is similar to the TCGA cohort. (B) Mean C-index is not significantly different between the validation and the additional external test set. (C) Accuracy is not significantly different between the validation and the external test set. (D) ROC and PR curves of the external test set. Ns, not significant. [file Image_3.jpeg]
