# Supplementary material for: Multimodal Deep Learning for Prognosis Prediction in Renal Cancer
Source: Front Oncol. 2021 Nov 24;11:788740. doi: 10.3389/fonc.2021.788740 (PMC8651560; doi:10.3389/fonc.2021.788740)
Supplement: Supplementary Table 1 — TCGA cohort. [file DataSheet_1.pdf]

Suppl_Table_1

Patient_ID	DSS_status	Months_of_DSS	5YSS	Grading	T-Stage	N-Stage	M-Stage	Count_Histo_L5	Count_Histo_L10	Count_Radiology	Entity_Radiology	Altered	VHL	PBRM1	SETD2	BAP1	MTOR	KDM5C	PCLO	ATM	STAG2	SPEN
TCGA-B0-4698	1	1.380806786	deceased	4	4	2	0	100	31	3	CT	0	0	0	0	0	0	0	0	0	0	0
TCGA-B0-4712	1	43.95568268	deceased	3	3	2	1	485	147	3	CT	1	1	1	1	0	0	0	0	0	0	0
TCGA-B0-4713	1	6.641023112	deceased	2	3	2	0	425	127	3	CT	0	0	0	0	0	0	0	0	0	0	0
TCGA-B0-4821	1	40.43791301	deceased	2	3	0	0	291	87	3	MRI	1	0	0	1	0	0	0	0	0	0	0
TCGA-B0-4833	1	78.44297597	living	2	1	0	0	381	116	3	CT	0	0	0	0	0	0	0	0	0	0	0
TCGA-B0-4839	0	53.88434099	NA	2	1	0	0	329	100	3	CT	0	0	0	0	0	0	0	0	0	0	0
TCGA-B0-4843	1	10.52043265	deceased	3	3	0	0	338	98	3	CT	0	0	0	0	0	0	0	0	0	0	0
TCGA-B0-4845	1	65.29243515	living	2	3	2	1	455	134	3	CT	0	0	0	0	0	0	0	0	0	0	0
TCGA-B0-4849	1	2.268468291	deceased	2	3	2	0	305	91	3	MRI	0	0	0	0	0	0	0	0	0	0	0
TCGA-B0-5077	0	43.29815564	NA	3	1	0	0	166	51	3	CT	1	1	0	0	0	0	1	0	0	0	0
TCGA-B0-5081	1	11.90123944	deceased	2	3	0	0	450	134	3	CT	1	1	1	1	0	0	0	0	0	0	0
TCGA-B0-5085	0	25.31479107	NA	3	3	0	0	198	56	3	MRI	1	1	1	0	0	0	0	0	0	0	0
TCGA-B0-5088	0	18.5093862	NA	3	1	0	0	374	107	3	CT	1	1	0	0	1	0	0	0	0	0	0
TCGA-B0-5099	1	15.94503074	deceased	3	3	2	0	337	98	3	CT	1	1	0	0	0	0	0	1	0	0	0
TCGA-B0-5106	0	52.53641056	NA	2	1	0	0	389	123	3	CT	1	1	0	0	0	0	1	0	0	0	0
TCGA-B0-5109	1	19.29841865	deceased	4	3	1	0	150	45	3	MRI	0	0	0	0	0	0	0	0	0	0	0
TCGA-B0-5110	0	66.04859125	living	2	1	0	0	221	68	3	CT	1	0	1	0	0	0	0	0	0	0	0
TCGA-B0-5115	0	52.73366867	NA	3	2	0	1	241	69	3	CT	1	1	1	0	0	0	0	0	0	0	0
TCGA-B0-5117	0	52.86517408	NA	2	1	2	0	530	153	3	CT	0	0	0	0	0	0	0	0	0	0	0
TCGA-B0-5121	0	48.82138278	NA	2	1	0	0	192	56	3	CT	1	0	1	1	0	0	0	0	0	0	0
TCGA-B0-5399	0	46.38853273	NA	2	1	0	0	722	214	3	CT	1	0	1	1	0	0	0	0	0	0	0
TCGA-B0-5696	0	85.77440247	living	4	3	0	0	219	66	3	CT	1	1	1	1	1	1	0	0	0	0	0
TCGA-B0-5697	0	86.46480587	living	2	1	0	0	235	73	3	CT	1	0	0	0	0	1	0	0	0	0	0
TCGA-B0-5698	0	119.3740343	living	3	1	0	0	270	85	3	CT	1	0	1	0	0	0	1	0	0	0	0
TCGA-B0-5702	0	71.40743663	living	2	1	0	0	266	80	3	CT	1	0	0	1	0	0	0	0	0	0	0
TCGA-B0-5703	0	73.84028668	living	3	1	0	0	236	70	3	CT	1	1	0	0	0	0	0	0	1	0	0
TCGA-B0-5706	0	105.3687083	living	2	3	0	0	378	115	3	CT	1	0	0	0	0	0	0	0	0	0	1
TCGA-B0-5707	0	123.089062	living	3	1	0	0	147	47	3	CT	0	0	0	0	0	0	0	0	0	0	0
TCGA-B0-5709	0	130.650623	living	3	3	2	0	221	66	3	CT	1	1	0	0	1	0	0	0	0	1	0
TCGA-B0-5711	0	131.1437683	living	3	3	2	0	289	84	3	CT	1	1	1	0	0	0	0	0	0	0	0
TCGA-B0-5712	0	89.48943025	living	3	2	0	1	88	26	3	CT	1	0	1	0	0	1	0	0	0	1	0
TCGA-B0-5812	0	126.0479337	living	3	1	2	0	243	75	3	CT	1	1	1	0	0	0	0	0	0	0	0
TCGA-B8-4146	0	16.79981589	NA	2	1	2	0	176	58	3	CT	0	0	0	0	0	0	0	0	0	0	0
TCGA-B8-4148	0	49.9720551	NA	3	1	0	0	300	92	3	CT	1	1	1	0	0	0	0	0	0	0	0
TCGA-B8-4151	0	42.7063813	NA	2	3	0	0	264	77	3	CT	1	0	1	0	1	0	0	0	0	0	0
TCGA-B8-4153	0	25.05178025	NA	3	3	2	0	696	211	3	CT	1	1	1	0	0	0	0	0	0	0	0
TCGA-B8-4154	0	45.36936582	NA	2	1	0	0	64	21	3	CT	1	1	1	0	0	0	0	0	0	0	0
TCGA-B8-4619	0	17.19433212	NA	2	1	0	0	80	23	3	CT	0	0	0	0	0	0	0	0	0	0	0
TCGA-B8-4620	0	25.54492554	NA	2	3	0	0	264	80	3	CT	1	1	1	1	0	0	0	0	0	0	0
TCGA-B8-4621	0	25.90656541	NA	3	1	0	0	451	129	3	MRI	0	0	0	0	0	0	0	0	0	0	0
TCGA-B8-4622	0	50.13643686	NA	3	3	0	1	384	115	3	CT	0	0	0	0	0	0	0	0	0	0	0
TCGA-B8-5158	0	40.04339679	NA	4	3	1	0	341	85	3	CT	1	0	1	0	0	0	0	0	0	0	1
TCGA-B8-5159	0	23.73672617	NA	3	1	0	0	70	20	3	CT	1	1	0	0	0	0	0	0	0	0	0
TCGA-B8-5162	0	1.183548673	NA	2	2	2	0	112	33	3	CT	1	1	0	0	0	0	0	0	0	0	0
TCGA-B8-5163	0	27.02436138	NA	3	3	0	0	187	46	3	CT	1	1	0	0	0	0	0	0	0	0	0
TCGA-B8-5164	0	0.854785153	NA	3	3	0	0	138	37	3	CT	0	0	0	0	0	0	0	0	0	0	0
TCGA-B8-5165	0	24.22987145	NA	2	1	0	0	172	45	3	CT	1	1	0	0	0	0	0	0	0	0	0
TCGA-B8-5545	0	50.13643686	NA	2	1	0	0	162	44	3	CT	0	0	0	0	0	0	0	0	0	0	0
TCGA-B8-5546	0	16.60255778	NA	2	1	0	0	204	63	3	MRI	1	0	0	0	0	0	0	1	0	0	0
TCGA-B8-5549	0	6.378012296	NA	3	1	0	2	287	88	3	CT	1	1	0	0	0	0	0	0	0	0	0
TCGA-B8-5550	0	48.52549561	NA	3	3	0	0	447	133	3	CT	1	0	0	0	1	0	0	0	0	0	0
TCGA-B8-5551	0	0.526021633	NA	3	1	0	0	223	67	3	CT	1	1	0	0	1	0	0	0	0	0	0
TCGA-B8-5552	0	34.38866423	NA	2	1	2	0	241	73	3	MRI	0	0	0	0	0	0	0	0	0	0	0
TCGA-B8-5553	0	14.30121314	NA	2	1	0	0	122	35	3	CT	0	0	0	0	0	0	0	0	0	0	0
TCGA-B8-A54D	0	27.28737219	NA	2	3	2	2	180	53	3	CT	1	0	0	0	0	0	0	0	0	1	0
TCGA-B8-A54E	0	29.884604	NA	3	1	2	2	413	122	3	CT	0	0	0	0	0	0	0	0	0	0	0
TCGA-B8-A54F	0	17.06282671	NA	2	1	2	2	542	160	3	CT	0	0	0	0	0	0	0	0	0	0	0
TCGA-B8-A54G	0	1.742446658	NA	3	1	2	2	253	71	3	CT	1	0	1	0	1	0	0	0	0	0	0

TCGA-BP-4164	0	32.61334122	NA	2	3	2	0	480	145	3	CT	1	1	1	0	0	0	0	0	0	0	0
TCGA-BP-4166	0	0.427392577	NA	3	3	0	0	219	66	3	CT	0	0	0	0	0	0	0	0	0	0	0
TCGA-BP-4167	0	89.35792485	living	2	3	2	0	90	29	3	CT	0	0	0	0	0	0	0	0	0	0	0
TCGA-BP-4169	1	23.04632278	deceased	2	2	0	0	591	175	3	CT	0	0	0	0	0	0	0	0	0	0	0
TCGA-BP-4170	0	77.02929283	living	2	1	0	0	536	160	3	MRI	0	0	0	0	0	0	0	0	0	0	0
TCGA-BP-4173	0	62.23493441	living	3	2	0	0	422	125	3	CT	0	0	0	0	0	0	0	0	0	0	0
TCGA-BP-4330	0	62.07055265	living	2	3	0	0	491	143	3	CT	0	0	0	0	0	0	0	0	0	0	0
TCGA-BP-4334	1	21.20524707	deceased	3	3	0	0	732	220	3	CT	0	0	0	0	0	0	0	0	0	0	0
TCGA-BP-4335	1	15.61626722	deceased	3	3	0	1	384	111	3	CT	0	0	0	0	0	0	0	0	0	0	0
TCGA-BP-4343	1	62.8595851	living	3	3	0	0	351	102	3	CT	0	0	0	0	0	0	0	0	0	0	0
TCGA-BP-4345	0	49.84054969	NA	3	3	0	0	198	62	3	CT	0	0	0	0	0	0	0	0	0	0	0
TCGA-BP-4346	0	49.0843936	NA	3	3	0	0	398	116	3	CT	0	0	0	0	0	0	0	0	0	0	0
TCGA-BP-4349	0	12.23000296	NA	2	1	2	0	275	81	3	CT	0	0	0	0	0	0	0	0	0	0	0
TCGA-BP-4351	0	31.89006148	NA	2	3	0	0	364	107	3	MRI	0	0	0	0	0	0	0	0	0	0	0
TCGA-BP-4352	1	11.3094651	deceased	4	3	0	1	449	134	3	CT	0	0	0	0	0	0	0	0	0	0	0
TCGA-BP-4354	1	33.99414801	deceased	4	4	1	1	450	135	3	CT	0	0	0	0	0	0	0	0	0	0	0
TCGA-BP-4355	1	31.33116349	deceased	4	3	2	0	377	114	3	CT	0	0	0	0	0	0	0	0	0	0	0
TCGA-BP-4759	0	77.98270704	living	2	1	2	0	354	107	3	CT	0	0	0	0	0	0	0	0	0	0	0
TCGA-BP-4760	0	77.62106717	living	2	1	2	0	254	78	3	MRI	0	0	0	0	0	0	0	0	0	0	0
TCGA-BP-4761	0	5.983496071	NA	4	4	1	0	479	137	3	MRI	0	0	0	0	0	0	0	0	0	0	0
TCGA-BP-4762	0	44.15294079	NA	3	1	2	0	34	12	3	CT	0	0	0	0	0	0	0	0	0	0	0
TCGA-BP-4763	0	41.75296709	NA	2	1	2	0	280	88	3	CT	0	0	0	0	0	0	0	0	0	0	0
TCGA-BP-4766	0	48.06522668	NA	3	1	2	0	359	107	3	CT	0	0	0	0	0	0	0	0	0	0	0
TCGA-BP-4768	0	13.15054082	NA	2	1	0	0	404	122	3	CT	0	0	0	0	0	0	0	0	0	0	0
TCGA-BP-4769	0	61.67603643	living	2	1	2	0	247	74	3	CT	0	0	0	0	0	0	0	0	0	0	0
TCGA-BP-4770	1	10.81631982	deceased	4	4	0	0	92	22	3	CT	0	0	0	0	0	0	0	0	0	0	0
TCGA-BP-4771	1	5.325969031	deceased	4	3	0	1	238	71	3	CT	0	0	0	0	0	0	0	0	0	0	0
TCGA-BP-4774	0	61.9719236	living	2	1	2	0	395	117	3	CT	0	0	0	0	0	0	0	0	0	0	0
TCGA-BP-4775	0	60.59111681	living	2	1	2	0	172	49	3	CT	0	0	0	0	0	0	0	0	0	0	0
TCGA-BP-4777	0	56.90896538	NA	3	1	2	0	192	60	3	CT	0	0	0	0	0	0	0	0	0	0	0
TCGA-BP-4781	0	68.38281224	living	3	1	2	0	301	92	3	CT	0	0	0	0	0	0	0	0	0	0	0
TCGA-BP-4782	0	11.63822862	NA	2	1	2	0	106	28	3	CT	0	0	0	0	0	0	0	0	0	0	0
TCGA-BP-4784	0	60.95275668	living	2	1	2	0	50	14	3	CT	0	0	0	0	0	0	0	0	0	0	0
TCGA-BP-4787	1	15.78064898	deceased	4	3	0	1	308	91	3	CT	0	0	0	0	0	0	0	0	0	0	0
TCGA-BP-4789	0	48.95288819	NA	2	1	2	0	97	31	3	CT	0	0	0	0	0	0	0	0	0	0	0
TCGA-BP-4790	0	36.52562712	NA	2	1	2	0	483	138	3	CT	0	0	0	0	0	0	0	0	0	0	0
TCGA-BP-4795	0	20.38333827	NA	2	1	0	0	68	23	3	MRI	0	0	0	0	0	0	0	0	0	0	0
TCGA-BP-4797	0	36.39412171	NA	3	3	0	0	322	97	3	CT	0	0	0	0	0	0	0	0	0	0	0
TCGA-BP-4799	1	37.24890686	deceased	3	3	0	0	246	76	3	CT	0	0	0	0	0	0	0	0	0	0	0
TCGA-BP-4801	0	36.95301969	NA	2	1	2	0	206	64	3	CT	1	0	1	0	0	0	0	0	1	0	0
TCGA-BP-4803	0	6.706775816	NA	3	3	2	0	169	49	3	CT	0	0	0	0	0	0	0	0	0	0	0
TCGA-BP-4804	0	47.96659763	NA	2	1	2	0	53	16	3	CT	0	0	0	0	0	0	0	0	0	0	0
TCGA-BP-4807	0	6.93691028	NA	3	1	2	0	150	46	3	CT	0	0	0	0	0	0	0	0	0	0	0
TCGA-BP-4960	0	71.40743663	living	3	2	0	0	349	106	3	CT	1	1	1	0	0	0	0	0	0	0	0
TCGA-BP-4962	0	58.68428839	NA	2	2	2	0	230	69	3	CT	1	1	0	0	1	0	0	0	0	0	0
TCGA-BP-4963	0	60.29522964	living	3	1	2	0	191	56	3	MRI	1	1	0	1	0	0	0	1	0	0	0
TCGA-BP-4964	0	61.2157675	living	2	1	0	0	153	42	3	CT	1	1	1	0	0	0	0	0	1	0	0
TCGA-BP-4965	0	61.51165467	living	2	1	2	0	71	20	3	CT	1	1	0	0	0	0	0	0	0	0	0
TCGA-BP-4967	0	6.739652168	NA	2	3	0	0	165	51	3	CT	1	1	0	0	0	0	0	0	0	0	0
TCGA-BP-4970	0	14.23546043	NA	3	1	0	0	223	63	3	CT	1	1	0	0	0	0	0	0	0	0	0
TCGA-BP-4971	0	48.88713548	NA	3	3	0	0	155	47	3	CT	1	1	0	0	0	0	0	0	0	0	0
TCGA-BP-4972	0	49.38028076	NA	3	3	2	0	250	75	3	CT	0	0	0	0	0	0	0	0	0	0	0
TCGA-BP-4973	0	45.50087122	NA	3	3	2	0	180	56	3	MRI	1	0	1	0	0	0	0	0	0	0	0
TCGA-BP-4975	0	47.11181247	NA	3	1	2	0	278	82	3	CT	1	0	1	0	0	0	0	0	0	0	0
TCGA-BP-4977	0	14.92586383	NA	3	1	2	0	128	38	3	CT	1	1	0	0	0	0	1	0	0	0	0
TCGA-BP-4982	0	33.33662097	NA	3	1	2	0	396	119	3	CT	1	1	1	0	0	0	0	0	0	0	0
TCGA-BP-4989	0	3.879409541	NA	3	3	0	0	144	43	3	CT	1	1	1	0	0	0	0	0	0	0	0
TCGA-BP-4992	0	16.47105237	NA	4	1	2	0	84	26	3	CT	0	0	0	0	0	0	0	0	0	0	0
TCGA-BP-4994	0	43.00226847	NA	3	1	2	0	465	139	3	CT	0	0	0	0	0	0	0	0	0	0	0

TCGA-BP-4999	0	41.62146168	NA	2	1	2	0	155	49	3	CT	1	1	0	0	0	0	0	0	0	0	0
TCGA-BP-5000	0	18.5093862	NA	3	1	2	0	237	72	3	CT	1	0	0	0	0	0	1	0	0	0	0
TCGA-BP-5001	0	38.69546635	NA	2	1	2	0	257	78	3	CT	1	0	0	0	0	1	0	0	0	0	0
TCGA-BP-5004	0	37.0187724	NA	3	1	2	0	354	105	3	CT	0	0	0	0	0	0	0	0	0	0	0
TCGA-BP-5007	0	37.47904133	NA	2	2	0	0	347	101	3	CT	1	0	0	0	0	1	0	0	0	0	0
TCGA-BP-5010	1	28.86543709	deceased	4	3	0	0	526	161	3	CT	1	0	0	1	0	0	0	0	0	1	0
TCGA-BP-5168	0	48.09810303	NA	2	1	0	0	366	108	3	CT	1	0	1	0	0	0	0	0	0	0	0
TCGA-BP-5169	0	6.345135944	NA	4	1	0	0	483	145	3	CT	1	1	1	1	0	0	1	0	0	0	0
TCGA-BP-5173	0	2.038333827	NA	2	1	2	0	511	149	3	MRI	1	0	0	0	0	0	0	0	1	0	0
TCGA-BP-5174	0	74.20192655	living	2	1	2	0	257	77	3	CT	1	1	0	0	0	0	0	0	0	0	0
TCGA-BP-5175	0	30.6407601	NA	3	1	2	0	166	51	3	CT	1	1	0	0	0	1	0	0	0	0	0
TCGA-BP-5178	1	62.8595851	living	4	4	0	1	295	85	3	MRI	1	1	0	1	0	0	0	0	0	0	0
TCGA-BP-5180	0	74.39918467	living	2	1	2	0	180	54	3	CT	1	1	1	0	0	0	0	0	0	0	0
TCGA-BP-5183	0	42.44337048	NA	3	3	2	0	115	34	3	CT	1	1	1	0	0	0	0	0	0	0	0
TCGA-BP-5184	0	37.24890686	NA	3	1	2	0	241	72	3	CT	1	1	1	0	0	0	0	0	0	0	0
TCGA-BP-5185	0	37.21603051	NA	3	1	2	0	423	126	3	MRI	1	1	1	0	0	0	0	1	0	0	0
TCGA-BP-5186	0	22.78331196	NA	2	1	0	0	359	108	3	CT	1	1	1	0	0	0	0	0	0	0	0
TCGA-BP-5187	0	13.34779893	NA	2	1	2	0	207	62	3	CT	1	1	1	0	0	0	0	0	0	0	0
TCGA-BP-5189	1	27.02436138	deceased	4	1	2	0	349	104	3	CT	1	0	1	0	0	0	1	0	0	0	0
TCGA-BP-5190	0	33.23799191	NA	3	1	2	0	283	84	3	CT	1	0	1	0	0	0	1	0	0	0	0
TCGA-BP-5191	0	31.79143242	NA	2	3	0	0	130	42	3	CT	1	0	1	1	1	0	0	1	0	1	0
TCGA-BP-5192	0	23.47371536	NA	2	1	2	0	271	80	3	CT	1	1	0	0	0	0	0	0	0	0	0
TCGA-BP-5194	0	13.41355163	NA	2	1	2	0	404	124	3	CT	1	0	1	0	0	0	0	0	0	0	0
TCGA-BP-5195	0	24.62438768	NA	2	1	2	0	200	61	3	CT	1	1	1	0	0	0	1	0	0	0	0
TCGA-BP-5196	0	33.46812638	NA	2	1	2	0	203	60	3	CT	1	1	0	0	0	0	0	0	0	0	0
TCGA-BP-5198	0	19.82444028	NA	3	3	0	0	379	109	3	MRI	1	1	1	1	0	0	0	0	0	0	0
TCGA-BP-5200	0	34.94756222	NA	4	2	2	0	89	27	3	CT	1	0	1	0	0	0	0	0	0	0	0
TCGA-BP-5201	0	31.26541079	NA	4	3	0	1	245	69	3	MRI	1	1	1	0	0	0	0	0	0	0	0
TCGA-BP-5202	0	0.953414209	NA	2	3	2	0	131	39	3	CT	1	0	0	0	0	0	1	0	0	0	0
TCGA-CJ-4635	0	46.55291449	NA	3	1	2	0	169	48	3	CT	0	0	0	0	0	0	0	0	0	0	0
TCGA-CJ-4636	0	63.25410133	living	3	3	0	0	266	78	3	CT	1	1	0	0	1	0	0	0	0	0	0
TCGA-CJ-4637	1	73.21563599	living	4	2	2	1	186	54	3	CT	1	1	0	0	1	0	0	0	0	0	0
TCGA-CJ-4641	1	54.60762074	deceased	4	3	2	1	242	77	3	CT	1	0	0	0	1	0	0	0	0	0	0
TCGA-CJ-4643	0	58.94729921	NA	3	2	0	0	206	58	3	CT	1	1	1	0	0	0	0	1	0	0	1
TCGA-CJ-4870	0	49.24877536	NA	2	3	2	0	202	58	3	CT	0	0	0	0	0	0	0	0	0	0	0
TCGA-CJ-4871	0	79.65940099	living	4	3	2	1	180	57	3	CT	0	0	0	0	0	0	0	0	0	0	0
TCGA-CJ-4873	0	74.26767926	living	3	3	0	0	279	82	3	CT	0	0	0	0	0	0	0	0	0	0	0
TCGA-CJ-4876	0	64.27326824	living	3	2	0	0	238	74	3	CT	0	0	0	0	0	0	0	0	0	0	0
TCGA-CJ-4884	0	57.82950324	NA	3	1	2	0	334	98	3	CT	0	0	0	0	0	0	0	0	0	0	0
TCGA-CJ-4885	0	113.4562909	living	3	3	2	1	363	108	3	CT	0	0	0	0	0	0	0	0	0	0	0
TCGA-CJ-4886	0	64.17463918	living	3	1	2	0	122	37	3	CT	0	0	0	0	0	0	0	0	0	0	0
TCGA-CJ-4887	1	30.6407601	deceased	3	3	2	1	131	40	3	CT	0	0	0	0	0	0	0	0	0	0	0
TCGA-CJ-4889	0	63.97738107	living	4	1	0	0	69	21	3	CT	0	0	0	0	0	0	0	0	0	0	0
TCGA-CJ-4891	0	26.92573232	NA	4	3	0	0	50	16	3	CT	0	0	0	0	0	0	0	0	0	0	0
TCGA-CJ-4892	0	50.00493145	NA	2	3	0	0	65	21	3	CT	0	0	0	0	0	0	0	0	0	0	0
TCGA-CJ-4893	0	24.65726403	NA	3	1	2	0	162	47	3	CT	0	0	0	0	0	0	0	0	0	0	0
TCGA-CJ-4894	1	27.64901207	deceased	3	3	0	0	377	113	3	CT	0	0	0	0	0	0	0	0	0	0	0
TCGA-CJ-4895	1	39.45162245	deceased	4	3	2	1	331	100	3	CT	0	0	0	0	0	0	0	0	0	0	0
TCGA-CJ-4897	0	109.8398922	living	3	4	2	0	288	84	3	CT	1	1	1	1	1	0	0	0	0	0	0
TCGA-CJ-4899	0	50.23506592	NA	2	1	2	0	98	30	3	CT	0	0	0	0	0	0	0	0	0	0	0
TCGA-CJ-4900	1	56.3500674	deceased	3	4	1	1	190	57	3	CT	1	1	0	0	0	0	0	0	0	0	0
TCGA-CJ-4901	0	47.67071046	NA	3	3	2	0	207	63	3	CT	1	1	1	1	1	0	0	0	0	0	1
TCGA-CJ-4902	0	49.9720551	NA	3	3	2	0	145	44	3	CT	1	1	0	0	0	0	0	0	0	0	0
TCGA-CJ-4903	0	51.28710918	NA	3	1	2	0	208	63	3	CT	1	1	1	0	0	0	0	0	0	0	0
TCGA-CJ-4904	0	108.5577144	living	3	3	0	1	369	109	3	CT	1	1	1	0	0	0	0	0	1	0	0
TCGA-CJ-4905	0	49.18302265	NA	2	1	2	0	195	59	3	CT	1	0	1	0	0	0	0	0	0	0	0
TCGA-CJ-4907	0	49.28165171	NA	3	3	2	0	301	92	3	CT	1	0	0	0	0	1	0	0	0	0	0
TCGA-CJ-4908	0	50.33369497	NA	2	1	2	0	79	24	3	CT	1	1	1	0	0	0	0	0	0	0	0
TCGA-CJ-4912	0	54.47611533	NA	3	2	2	0	181	55	3	CT	1	1	1	0	0	0	0	1	0	0	0

TCGA-CJ-4913	1	38.56396094	deceased	4	3	2	0	419	124	3	CT	1	0	1	0	0	1	0	0	1	0	0
TCGA-CJ-4916	0	45.13923135	NA	3	3	2	0	371	108	3	CT	1	0	1	0	0	0	0	0	0	1	0
TCGA-CJ-4918	1	3.05750074	deceased	4	3	0	1	113	32	3	CT	1	0	0	0	0	0	0	1	0	0	0
TCGA-CJ-4920	0	4.569812934	NA	2	1	2	0	174	55	3	CT	1	0	1	1	0	0	0	0	0	0	0
TCGA-CJ-4923	1	18.80527337	deceased	4	3	2	1	235	71	3	CT	1	1	1	1	1	0	0	0	0	0	0
TCGA-CJ-5671	0	131.0780156	living	3	1	2	0	91	29	3	CT	1	0	1	1	0	0	0	0	0	0	0
TCGA-CJ-5672	0	71.99921097	living	3	1	2	0	68	22	3	CT	1	1	0	0	0	0	0	0	0	0	0
TCGA-CJ-5675	0	129.4013216	living	3	2	2	0	94	26	3	CT	1	0	1	0	0	0	0	0	0	0	0
TCGA-CJ-5677	1	25.7093073	deceased	4	3	2	1	85	26	3	CT	1	1	0	0	0	0	0	0	0	0	0
TCGA-CJ-5678	1	18.87102607	deceased	3	2	0	1	86	29	3	CT	1	0	1	1	0	0	0	0	0	0	0
TCGA-CJ-5679	1	22.32304304	deceased	4	3	2	0	185	59	3	CT	1	0	1	0	0	1	0	0	0	0	0
TCGA-CJ-5680	1	25.24903837	deceased	4	3	2	1	104	34	3	CT	1	1	0	0	1	0	0	0	0	0	0
TCGA-CJ-5681	1	18.14774633	deceased	3	3	2	1	18	5	3	CT	1	0	1	1	1	0	0	0	0	0	0
TCGA-CJ-5683	0	62.103429	living	3	1	2	0	37	11	3	CT	1	0	1	0	0	0	0	0	0	0	0
TCGA-CJ-5686	0	67.00200546	living	3	1	2	0	106	31	3	CT	1	1	0	0	0	0	0	0	1	0	0
TCGA-CJ-6027	0	118.8480126	living	4	1	2	0	184	56	3	CT	1	1	1	0	0	1	0	0	0	0	0
TCGA-CJ-6028	1	53.42407207	deceased	4	3	2	1	178	50	3	CT	1	1	1	1	1	0	0	0	0	0	0
TCGA-CJ-6030	0	75.58273334	living	3	1	0	0	212	61	3	CT	1	1	1	0	0	0	0	0	0	0	0
TCGA-CJ-6031	0	62.66232699	living	3	1	2	0	173	53	3	MRI	0	0	0	0	0	0	0	0	0	0	0
TCGA-CJ-6033	1	7.364302857	deceased	4	3	0	1	281	84	3	CT	1	1	1	0	0	0	0	0	0	0	0
TCGA-CZ-4857	1	47.07893612	deceased	3	3	0	1	125	31	3	MRI	0	0	0	0	0	0	0	0	0	0	0
TCGA-CZ-4858	1	69.20472104	living	4	2	2	0	90	23	3	CT	0	0	0	0	0	0	0	0	0	0	0
TCGA-CZ-4859	0	58.7500411	NA	2	1	0	0	100	24	3	CT	1	1	0	0	0	0	0	0	0	1	1
TCGA-CZ-4860	1	6.77252852	deceased	4	4	2	1	175	48	3	MRI	0	0	0	0	0	0	0	0	0	0	0
TCGA-CZ-4861	1	14.66285301	deceased	2	2	2	0	62	16	3	CT	0	0	0	0	0	0	0	0	0	0	0
TCGA-CZ-4862	0	107.5385475	living	2	1	2	0	76	18	3	CT	0	0	0	0	0	0	0	0	0	0	0
TCGA-CZ-4863	0	63.38560673	living	3	3	0	0	108	24	3	CT	0	0	0	0	0	0	0	0	0	0	0
TCGA-CZ-4866	0	107.4070421	living	3	1	2	0	134	35	3	CT	1	0	0	0	1	1	0	0	0	0	0
TCGA-CZ-5451	0	63.41848309	living	3	2	0	0	266	80	3	CT	1	0	1	0	0	0	0	0	1	0	0
TCGA-CZ-5452	0	58.8157938	NA	2	2	0	0	250	74	3	CT	1	0	0	0	1	0	0	0	0	0	0
TCGA-CZ-5454	1	23.73672617	deceased	2	2	0	1	324	97	3	MRI	1	0	1	0	0	0	0	0	0	0	0
TCGA-CZ-5455	1	18.44363349	deceased	4	3	2	1	189	59	3	MRI	1	0	1	0	0	0	0	0	0	0	0
TCGA-CZ-5460	0	94.45375941	living	3	3	2	1	269	80	3	MRI	1	1	1	1	1	0	0	0	0	0	0
TCGA-CZ-5462	1	10.22454548	deceased	3	1	2	1	245	80	3	CT	0	0	0	0	0	0	0	0	0	0	0
TCGA-CZ-5464	0	69.96087714	living	2	3	2	1	493	153	3	MRI	1	1	1	1	1	0	0	0	0	0	0
TCGA-CZ-5466	0	22.52030115	NA	2	3	2	0	674	203	3	MRI	1	0	1	0	0	0	0	0	0	1	0
TCGA-CZ-5468	1	1.93970477	deceased	4	3	2	1	409	125	3	CT	1	1	0	0	0	0	0	1	0	0	0
TCGA-CZ-5984	0	67.95541967	living	3	1	0	0	355	109	3	CT	1	1	0	0	0	0	0	0	0	0	0
TCGA-CZ-5985	0	65.65407502	living	2	2	0	0	298	91	3	CT	1	1	0	0	1	0	0	1	0	0	0
TCGA-CZ-5987	1	14.62997666	deceased	2	3	2	1	237	68	3	MRI	1	1	1	0	0	1	0	0	0	0	0
TCGA-CZ-5988	0	22.78331196	NA	2	1	0	0	189	58	3	CT	0	0	0	0	0	0	0	0	0	0	0
TCGA-CZ-5989	0	62.62945064	living	2	2	0	0	181	53	3	MRI	1	1	1	0	0	0	0	0	0	0	0
TCGA-DV-5565	0	43.69267186	NA	2	1	2	0	103	30	3	CT	0	0	0	0	0	0	0	0	0	0	0
TCGA-DV-5566	0	45.96114015	NA	2	1	2	2	280	84	3	CT	1	0	1	0	0	0	0	0	0	0	0
TCGA-DV-5567	0	65.88420949	living	2	1	2	0	172	50	3	CT	1	1	0	0	0	0	0	0	0	0	0
TCGA-DV-5574	0	66.27872571	living	2	1	2	0	230	68	3	CT	0	0	0	0	0	0	0	0	0	0	0
TCGA-DV-5575	0	56.84321268	NA	2	1	2	0	163	50	3	CT	1	1	0	0	0	0	0	0	0	0	0
TCGA-DV-5576	1	23.90110793	deceased	2	1	2	0	114	34	3	CT	0	0	0	0	0	0	0	0	0	0	0
TCGA-DV-A4VX	1	53.45694842	deceased	4	3	0	2	414	122	3	MRI	1	1	0	1	0	0	1	0	0	0	1
TCGA-DV-A4VZ	0	11.9998685	NA	2	1	2	2	109	36	3	MRI	0	0	0	0	0	0	0	0	0	0	0
TCGA-DV-A4W0	0	81.20458954	living	2	1	2	2	739	223	3	CT	1	0	1	0	1	0	0	0	0	0	0
TCGA-G6-A5PC	1	7.956077194	deceased	4	1	0	1	305	91	3	CT	1	0	0	0	1	0	0	0	0	0	0
TCGA-G6-A8L6	1	10.29029819	deceased	3	2	2	2	137	41	3	CT	1	1	1	1	0	0	0	0	0	0	0
TCGA-G6-A8L7	0	70.1252589	living	3	1	0	2	432	128	3	CT	1	0	1	0	0	0	0	0	0	0	0
