# Supplementary material for: Multimodal Deep Learning for Prognosis Prediction in Renal Cancer
Source: Front Oncol. 2021 Nov 24;11:788740. doi: 10.3389/fonc.2021.788740 (PMC8651560; doi:10.3389/fonc.2021.788740)
Supplement: Supplementary Table 2 — Additional external test cohort (the Mainz cohort). [file DataSheet_2.pdf]

Suppl_Table_2

Patient_ID	DSS_Status	Months_of_DSS	5YSS	Grading	T-Stage	N-Stage	M-Stage	Count_Histo_L5	Count_Histo_L10	Count_Radiology	Entity_Radiology
ANO_00264	0	71	living	G1	pT1b	pNx	Mx	240	89	3	CT
ANO_00777	0	76	living	G3	pT3a	pNx	Mx	82	28	3	CT
ANO_00874	0	62	living	G1	pT1a	pNx	Mx	48	36	3	CT
ANO_01464	1	1	deceased	G3	pT3a	pN0	pM1	192	73	3	CT
ANO_01958	1	12	deceased	G3	pT4	pN0	Mx	89	36	3	CT
ANO_03634	1	41	deceased	G3	pT3a	pN1	Mx	52	20	3	MRI
ANO_03848	0	70	living	G1	pT1a	pNx	Mx	230	88	3	CT
ANO_04251	0	98	living	G1	pT1a	pNx	Mx	81	32	3	CT
ANO_05409	0	74	living	G3	pT1a	pNx	Mx	130	48	3	CT
ANO_07772	0	66	living	G2	pT3a	pNx	Mx	54	30	3	CT
ANO_07865	1	1	deceased	G2	pT3a	pN0	pM1	157	58	3	CT
ANO_08613	0	62	living	G3	pT1b	pNx	Mx	146	56	3	CT
ANO_08736	0	91	living	G2	pT1a	pNx	Mx	197	73	3	CT
ANO_09164	0	65	living	G2	pT1a	pNx	Mx	60	30	3	CT
ANO_09225	0	62	living	G2	pT1a	pNx	Mx	52	32	3	CT
ANO_09404	1	0	deceased	G4	pT4	pN0	pM1	65	23	3	CT
ANO_09416	0	89	living	G1	pT1b	pNx	Mx	109	37	3	CT
ANO_09579	1	43	deceased	G3	pT3a	pN0	Mx	153	55	3	CT
